# Teaching history taking to medical students: a systematic review

**DOI:** 10.1186/s12909-015-0443-x

**Published:** 2015-09-28

**Authors:** Katharina E. Keifenheim, Martin Teufel, Julianne Ip, Natalie Speiser, Elisabeth J. Leehr, Stephan Zipfel, Anne Herrmann-Werner

**Affiliations:** 1 Department for Psychosomatic Medicine and Psychotherapy, University Hospital of Tuebingen, Osianderstr. 5, 72076 Tübingen, Germany; 2 Clinical Associate Professor of Family Medicine, Associate Dean of Medicine, Brown University, Providence, RI USA; 3 Dean of Medical Education, Medical Faculty, University of Tuebingen, Tuebingen, Germany

**Keywords:** Undergraduate medical education, Medical students, History taking, Medical history, Medical interview, Interview skills

## Abstract

**Background:**

This paper is an up-to-date systematic review on educational interventions addressing history taking. The authors noted that despite the plethora of specialized training programs designed to enhance students‘ interviewing skills there had not been a review of the literature to assess the quality of each published method of teaching history taking in undergraduate medical education based on the evidence of the program’s efficacy.

**Methods:**

The databases PubMed, PsycINFO, Google Scholar, opengrey, opendoar and SSRN were searched using key words related to medical education and history taking. Articles that described an educational intervention to improve medical students’ history-taking skills were selected and reviewed. Included studies had to evaluate learning progress. Study quality was assessed using the Medical Education Research Study Quality Instrument (MERSQI).

**Results:**

Seventy-eight full-text articles were identified and reviewed; of these, 23 studies met the final inclusion criteria. Three studies applied an instructional approach using scripts, lectures, demonstrations and an online course. Seventeen studies applied a more experiential approach by implementing small group workshops including role-play, interviews with patients and feedback. Three studies applied a creative approach. Two of these studies made use of improvisational theatre and one introduced a simulation using Lego® building blocks. Twenty-two studies reported an improvement in students’ history taking skills. Mean MERSQI score was 10.4 (range 6.5 to 14; SD = 2.65).

**Conclusions:**

These findings suggest that several different educational interventions are effective in teaching history taking skills to medical students. Small group workshops including role-play and interviews with real patients, followed by feedback and discussion, are widespread and best investigated. Feedback using videotape review was also reported as particularly instructive. Students in the early preclinical state might profit from approaches helping them to focus on interview skills and not being distracted by thinking about differential diagnoses or clinical management. The heterogeneity of outcome data and the varied ways of assessment strongly suggest the need for further research as many studies did not meet basic methodological criteria. Randomized controlled trials using external assessment methods, standardized measurement tools and reporting long-term data are recommended to evaluate the efficacy of courses on history taking.

## Background

In the course of his or her professional life, a clinician will conduct between 100,000 and 200,000 patient interviews [1, 2]. The medical interview is the most common task performed by physicians. Thus, for good reason, Engel and Morgan called it “the most powerful and sensitive and most versatile instrument available to the physician” [3]. Scientific discoveries and technological innovations of the last decades fundamentally changed diagnostics and treatment of diseases. Imaging studies and laboratory tests seem crucial for an accurate diagnosis, all the more in times of multidisciplinary treatments and overall availability of instrument-based examinations. But neither scientific nor technological advances in medicine have changed the fact that a physician’s core clinical skills are interpersonal [4–6]. Interview skills contribute significantly to problem detection, diagnostic accuracy, patient and physician satisfaction, patient adjustment to stress and illness, patient recall of information, patient adherence to therapy and patient health outcomes [7–11]. Accuracy of diagnoses and the establishment of a good physician-patient relationship depend on effective communication within the medical interview [12, 13]. By the medical history, physicians garner 60–80 % of the information that is relevant for a diagnosis [13–17] and the history alone can lead to the final diagnosis in 76 % [13].

There are different definitions and models of history taking in the international literature, suggesting a limited shared understanding of the medical interview. Several statements and checklists try to define what qualifies a medical interview as “good” and come to divergent results. One reason might be that history taking is highly contextual, depending on situation, patient and physician attributes, cultural characteristics and other factors. For example, a “good” medical interview in an emergency ward would differ distinctly from a “good” first interview in a psychiatric medical practice. Several authors refer to the “three-function model” [18] that highlights gathering data (1), responding to patients’ emotions (2) and educating patients and influencing their behaviour (3) as main functions of the medical interview. Each function is served by a separate set of skills. Other models focus on risk assessment, collection of data to make a diagnosis and assessment of patients’ available support system [19] as main tasks within the medical interview. The “five step model” [20] links physicians’ patient-centred skills with a more focused proceeding within the interview. Other models emphasise patient-centeredness even more, describing an equal exchange of information and shared decision-making [21, 22]. Despite this heterogeneity, there seems to be an agreement that in a “good” medical interview, patient-centered techniques must at least complement the traditional clinician-centred focused questioning style.

Being a successful communicator has long been seen as part of the “art” of medicine, implying that communication skills were a natural gift with which one was or was not born [23]. However, some researchers described that basic communication skills deteriorate during medical education if they are not particularly activated and practised [24, 25]. Students’ psychosocial interviewing skills especially seem to decline without targeted interventions [7, 19, 25]. This has often been associated with students’ growing medical knowledge and concentration on clinical reasoning and diagnostic skills. On the other hand, many studies have shown that students, having passed specialized history taking skills training, ask relevant questions and structure their interviews well. They are better at responding appropriately to patients’ verbal and non-verbal cues [26] as well as being able to elicit greater quantity and quality of information [27, 28].

History taking and communication skills programmes have become cornerstones in medical education over the past 30 years and are implemented in most US [6],Canadian [8], German [29] and UK [30] medical schools. National accreditations and expert panel consensus guidelines have stressed the importance of educational interventions addressing history taking [31, 32]. Today, it is a proven fact that interview skills can be taught if effective methods are used. Even 25 years ago, articles and consensus statements outlined the assumed essential elements of effective interview skills courses [33, 34], despite not having much experiential evidence for their recommendations. Since then, many studies investigated the effectiveness of a multitude of different educational methods for teaching history taking. But there is still an uncertainty about: which of these methods are particularly effective; when in the curriculum they should be implemented; or which method is especially helpful for certain subgroups, for example, male or female students or not being a native speaker. In view of this uncertainty, the present systematic review of the literature has been undertaken to collect the currently reported knowledge in the field of teaching history taking in order to make recommendations for curriculum planners, medical teachers and future investigators.

### Review objectives

This review aims to answer the following questions: (1) What interventions to teach history taking to medical students exist? (2) How has the effectiveness of these interventions been measured? (3) What is the quality of evidence for these interventions?

## Method

### Information sources and search

This review process was conducted according to the PRISMA statement [35, 36]. The databases PubMed, PsycINFO and GoogleScholar were searched for articles published between January 1990 and June 2014. Hand searches were performed in the reference lists of the search results. Additionally, the “grey literature” databases opengrey, opendoar and SSRN were searched.

Search terms were related to history taking and medical education, using combinations of the following: *medical history taking, history-taking, medical communication, medical interview, anamnesis, medical students, medical education and teaching*. Search was narrowed to titles and abstracts and terms were searched as MeSH-Terms in PubMed. It was ensured that the search terms captured the previously published reviews [37, 38] and all relevant studies included in these reviews.

### Underlying definition of “history-taking”

The authors of this review understand “history-taking” as a way of eliciting relevant personal, psychosocial and symptom information from a patient with the aim of obtaining information useful in formulating a diagnosis and providing medical care to the patient. The medical interview is seen as an encounter between physician and patient, both contributing to the results.

### Inclusion criteria

Articles were included if the following criteria were met:

- Description of an educational intervention concerning history taking: This review investigates (introductory) workshops teaching history-taking in general, considering content, completeness, verbal and non-verbal interviewing techniques and rapport.
- Evaluation of learning progress (at least a self-evaluation of students)
- Reporting on undergraduate medical education (i.e. “medical students”)
- Publication dates between January 1, 1990 and June 30, 2014
- English- or German-language articles

We also included articles that described teaching units addressing other clinical skills (e.g. physical examination or clinical reasoning) in addition to history taking if intervention and outcomes concerning history taking were reported in detail and separately from the results regarding the other objectives.

### Exclusion criteria

The following results were excluded in this review:

- Teaching units concerning only specific aspects of the medical history (e.g. taking a sexual history or an occupational history). Specific aspects of the medical interview are usually taught later in medical education and after an introductory course in medical interviewing has taken place, which is why interventions with regard to these specific aspects were excluded in this review.
- Teaching units addressing communication skills in general, patient-centred behaviour or empathy without regard to history taking
- Articles describing only the assessment of interview skills without describing a teaching unit
- Articles with no measured outcome at all, e.g. project descriptions with course evaluation only and without any assessment of learning progress

### Article selection and data collection

**Fig. 1:**
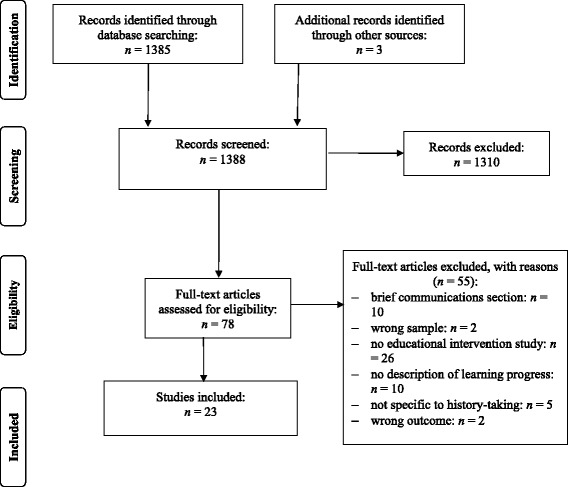
Flow chart of the literature search and study selection process

The literature search yielded 1254 potential publications on teaching units addressing history taking for medical students (see flowchart in Fig. 1 for complete search and study selection strategy). Following an initial review for relevancy by title and abstract (KEK and NS) and removal of duplicate results, 78 studies were left for full-text review, of these, 23 studies finally met the inclusion criteria. Interrater reliability was excellent with к = 0.84. In case of differing judgement, EJL was consulted as independent evaluator.

Relevant data was extracted from the included articles using an *a priori* developed data extraction form composed for this review (KEK, NS). Data extraction fields included (1) authors and year of publication, (2) description of study design and (3) participants, (4) description of the educational intervention, (5) assessment techniques and measurement tools, (6) reported change in history taking ability and (7) MERSQI score. Discussion with EJL resolved differences in data extraction.

### Quality assessment

Study quality was considered using the Medical Education Research Study Quality Instrument (MERSQI), a tool developed especially to assess educational studies [39]. The 10-item scale (possible range 5 to 18) surveys the following domains: study design, sampling, type of data, validity of the evaluation instrument, data analysis and outcomes. Patient or health care outcomes are assessed higher than students’ satisfaction, attitudes or opinions. The MERSQI domains are very similar to the required methodical standards that Sanson-Fisher suggested for educational studies [40]. Neither the authors of the MERSQI scale nor Sanson-Fisher and colleagues defined a cut-off value to differ methodically “good” studies from “less good studies”.

## Results

This systematic review includes 23 studies. Table 1 describes these studies in detail, reporting basic data concerning study design and participants, teaching methods and training procedures, assessment of learning progress, use of measurement tools and the calculated MERSQI score of the study (see Table 1).

**Table 1:** Characteristics of 23 studies of educational interventions concerning history-taking skills

Source	Study design	Participants	Educational intervention	Assessment technique and measurement tool	Improvement in history-taking ability reported?	MERSQI Score
Instructional (traditional) approaches						
Focus scripts						
Peltier et al. 2007 [44]	Randomized, two-group post-test	*n* = 60 clinical medical students	Focus Scripts	Students’ write-ups were scored by a blinded rater	Yes	12,5
Videotape review: Communication benchmarks						
Losh et al. 2005 [45]	Single-group post-test	*n* = 180 preclinical medical students	One 2-hour teaching session including an introduction of communication benchmarks and a video demonstration of short scenarios contrasting “ok” with “better” communication skills	Course evaluation questionnaire	Yes	6,5
Online course						
Wiecha et al. 2003 [46]	Single-group, pre-post comparison	*n* = 10 preclinical medical students	Four weeks online elective course including video demonstrations, text modules, a moderated, asynchronous discussion board and written personal feedback	Questionnaire on self-reported knowledge; qualitative analysis of interviews, focus groups and student course postings.	Yes	11
Experiental approaches						
Small group workshops including role-play and feedback						
Mukohara et al. 2004 [41]	Non-randomized, two-group post-test	*n* = 105 clinical medical students	Two-day, small group seminar, including role-play, videotape review, feedback and discussion	OSCE station; communication skills rated by two trained observers	Yes in one skill, tendency notable in 15 other skills	11,5
Evans et al. 1993 [27]	Non-randomized, two-group post-test	*n* = 106 preclinical medical students	Programme of lectures and skills workshops	Assessment of videotaped interviews with real patients by two independent, trained raters using the HTRS	Yes	11,5
Small group workshops including simulated patients						
von Lengerke et al. 2011 [51]	Single-group, pre-post comparison	*n* = 267 preclinical medical students	Seven 4-hour sessions in small groups, training program with lecture, self-study, (videotaped) role-play and SP interviews	Self-evaluation questionnaire on communication skills, course evaluation	Yes	8,5
Ozcakar et al. 2009 [42]	Randomized, two-group, pre-post comparison	*n* = 52 preclinical medical students	Two videotaped SP interviews and (visual/verbal) feedback by trainer	Self-assessment, assessment by trained observers using a checklist	Yes	13,5
Hulsman et al. 2009 [49]	Single-group post-test	*n* = 331 preclinical medical students	Seven small group sessions including SP interviews, videotape review, written self-evaluations, peer-feedback and discussion	Rating of students’ reflections by trained observers; evaluation questionnaire	Yes	9
Nestel& Kidd 2003 [50]	Randomized, two-group post-test	*n* = 40 preclinical medical students	One 3-hour session, including SP interviews, feedback and videotape review. Small groups facilitated either by peer tutors or by medical teachers	Written course evaluation questionnaire; self-assessment; rating by SPs and trained assessors	Yes	13
Yedidia et al. 2003 [52]	Randomized, two-group, pre-post comparison	*n* = 293 clinical medical students	Demonstration of interviewing skills, SP interviews, feedback and self-reflection	OSCE station, communication skills rated by SPs	Yes	13,5
Fortin et al. 2002 [7]	Single-group post-test	*n* = 127 preclinical medical students	Two half-day workshops including a mini lecture, demonstration by faculty, role-play, SP interviews and discussion	Course evaluation questionnaire; free-text on what students learned from the workshop	Yes	7
Utting et al. 2000 [43]	Randomized, single-blinded, three-group post-test comparison	*n* = 111 clinical medical students	Two 4-week basic skills courses including small group activities, discussion, role-play and SP interviews compared with a 10-week course including mainly lectures and instructions	Evaluation of videotaped SP interviews by two independent observers using IGS and CSS	No	12,5
Eoaskoon et al. 1996 [48]	Non-randomized, three-group post-test	*n* = 115 clinical medical students	Theoretical sessions, then division into three groups: (1) SP interview and feedback, (2) role-play in front of the group and feedback, (3) role play within the group and feedback	Course evaluation, assessment of students’ interviews by tutors	Yes	8
Battles et al. 1992 [47]	Two-group post-test	*n* = 358 preclinical medical students	Small group sessions one-half day every 2 weeks using lectures, SP interviews, feedback and discussion	OSCE using brief SP encounters and writing stations	Yes	8
Kraan et al. 1990 [24]	Modified cross-sectional study	*n* = 563 preclinical and clinical medical students from five different academic years	Six-year undergraduate curriculum teaching communication skills using small group sessions including SP interviews, videotape review, feedback and discussion	Assessment of live SP interviews by trained observers using the MAAS	Yes	10,5
Using virtual patients						
Vash et al. 2007 [53]	Randomized, two-group post-test	*n* = 48 clinical medical students	Fourteen 1-hour sessions in a computer lab working through virtual patients in small groups	Written examination	Yes	11
Small group workshops including real patients						
Fischer et al. 2005 [54]	Single-group, pre-post comparison	*n* = 154 clinical medical students	9 weekly 2-hour small group sessions including role-plays, SP interviews and videotaped interviews with real patients, each followed by feedback	Pre and post self-assessment by students using a 1–6 point scale; OSCE stations where skills were rated by SP and trained observer	Yes	10,5
Windish et al. 2005 [10]	Randomized, two-group pre-post comparison	*n* = 121 preclinical medical students	Six weekly 3-hour small group sessions including brief lecture, short video highlighting certain skills and role-play with feedback	Assessment of student performance by trained SPs using a checklist; course evaluation	Yes	13,5
Evans et al. 1996 [26]	Randomized, two-group, pre-post comparison	*n* = 60 clinical medical students	Training programme including lectures, comprehensive notes and workshops with role-plays, videotaping of real patients and SPs and discussions in small groups	Rating of videotaped SP interviews by trained, blinded observers using the MIRS	Yes	12,5
Novack et al. 1992 [9]	Single-group, pre-post comparison	*n* = 60 preclinical medical students	Two initial lecture demonstrations, then 12 weekly 2-hour sessions in small groups including role-plays, interviews with patients and discussions.	Videotaped SP interviews rated by blind reviewers using ISIE; Brief questionnaire for students’ self-evaluation of progress	Yes	14
Creative approaches						
Improvisational theatre						
Shochet et al. 2013 [56]	Single-group post-test	*n* = 38 preclinical medical students	Four weekly 2-hour sessions, improvisational theatre	Online course evaluation, qualitative analysis of students’ comments	Yes	6,5
Watson 2011 [55]	Single-group post-test	*n* = 116 preclinical medical students	Five weekly 2-hour sessions in small groups, improvisational theatre	Qualitative analysis of course evaluations, self-report questionnaire on acquired skills	Yes	7
Lego® simulation						
Harding& D’Eon 2001 [57]	Single-group, pre-post comparison	*n* = 57 preclinical medical students	Two-hour session including interactive lecture and a Lego simulation	Survey, information recall, qualitative analysis of focus groups	Yes	7

*SP* simulated patient, *OSCE* Objective Structured Clinical Examination, *IGS* Information Gathering Scale, *CSS* Communication Skills Scale, *MIRS* Medical Interview Rating Scale, *HTRS* History-Taking Rating Scale, *ISIE* International Analysis System for Interview Evaluation, *MAAS* Maastricht History-Taking and Advice Checklist

### Study characteristics

The study design of the 23 finally selected articles was heterogeneous. There were randomized, two-group, pre-post comparisons (*n* = 4) as well as randomized and non-randomized two-group post-tests (*n* = 6). Five studies were single-group pre-post comparisons and five were single-group post-test evaluations only. Two were modified cohort controlled studies and one a non-randomized, three-group post-test. Of those studies reporting the duration of their educational interventions, the shortest intervention took two hours and the longest took seven 4-hour sessions (28 h).

### Outcome measures

Assessment methods and measurement tools, much like the study designs, also were very heterogeneous. In eight studies [9, 10, 24, 26, 28, 41–43] out of 22, trained observers assessed an interaction between a student and a simulated patient (SP) using a standardized history taking measurement tool. Seven of the applied scales were specific to history taking, but only one had a proven reliability and validity and all of them had been developed especially to assess the published intervention. The remaining 15 studies used either non-validated, self-report questionnaires developed by the respective study investigators, course evaluation questionnaires or qualitative analyses of students’ comments. One of the studies used a written examination; one used focus groups. Twenty-two studies out of 23 found positive effects of their educational interventions on students’ history-taking skills. For a full overview of the results see Table 1.

### Study quality

The mean MERSQI score for the 23 included studies was 10.36 (SD 2.65) [39]. The range was from 6.5 to 14 (possible range 5 to 18). Scores were limited especially by: deficiencies in the field of study design (ex: no control group, missing baseline measurements or lack of randomization); by missing validity of the outcome measurement tools; and by measurement of students’ attitudes or skills rather than by patient or health care outcomes.

### Interventions

#### Instructional (traditional) approaches

##### Focus scripts

Students in the multi-institutional RCT of Peltier [44] received “focused history and physical exam scripts” (Focus Scripts). The authors developed one generic acute patient script template and one template for a focused chronic illness history. The organizational structure of the scripts was aimed to support students’ collection of data on any symptom. Students’ written progress notes were scored by a blind rater using a standardized scale. Five of 11 variables were statistically higher in the group that learned with the focused scripts. These included history taking, clarity of diagnosis and overall score. This intervention focuses on content and completeness of the medical interview and does not take verbal or non-verbal interview skills into account.

#### Videotape review: Communication benchmarks

Losh [45] held a lecture introducing communication benchmarks for inpatient history and then showed short videotaped scenarios that illustrated segments of a student history, contrasting an acceptable version of communication with a better version. The better version demonstrated the appropriate benchmarked skills. The scenarios were used in teaching sessions to help students identify effective communication techniques within the medical interview. Participants were medical students doing their first medical interview. After the sessions, 76 % of the students felt that this design helped them to understand the introduced communication benchmarks and 92 % felt that the videotape helped to point out subtle communication issues that might otherwise have been missed. The intervention imparted both knowledge about content and structure of the medical interview and particular communication skills.

#### Online course

Wiecha [46] reported on an online course developed to teach the cognitive basis for interviewing skills. The authors provided video demonstrations of patient interviews, text modules presenting communication concepts (not further clarified by the authors) and a moderated, asynchronous discussion board asking students to post their observations. The authors addressed questioning techniques, affect and nonverbal cues, eliciting the cardinal features of a symptom, and stages and transitions. Students received individual feedback on their participation and performance by personal e-mail. They reported improvement in self-awareness, increased understanding of interviewing concepts and benefits of online learning. Self-reported knowledge scores also increased significantly.

#### Experiential (“learning by doing”) approaches

##### Small group workshops including role-play and feedback

In two studies [28, 41], students participated in small group workshops practising history taking by role-play. Feedback was provided by facilitator and group members. Evans [28] implemented a specialized history-taking training programme consisting of lectures and skills workshops. Trained students were significantly more efficient on all areas covered by the applied scale (commencement of the interview, problem processing, communication, summary and overall effectiveness). In a non-randomized, controlled study, Mukohara [41] implemented a 2-day seminar on communication process skills and content aspects of the medical interview. Learning activities were a trigger videotape critique followed by role-play with videotape review and feedback by facilitator and group. The authors found an improvement for students’ ability to assess “how the illness affects the patient’s life”. No differences were observed between intervention group and waiting control group in the other 15 core communication skills.

#### Small group workshops including simulated patients

Ten studies [7, 24, 28, 42, 43, 47–52] reported on interventions using simulated patients (SP). SP interviews were conducted by one of the participating students and were usually combined with a feedback session and discussion. Feedback was given by the group and/or the facilitator. SP interviews in these workshops were often supplemented by lectures, demonstrations, small group exercises including role-play and self-reflection. Battles [47] used SPs with abnormal medical histories to demonstrate pathology. Utting [43] compared two skills courses using an active “learning by doing” approach with one course and applying instructional methods in the other. The authors found no differences in students’ interview skills, which were assessed using standardized scales. Eoaskoon [48] conducted a three-group post-test. SP interview and feedback (1) were compared with role-play and feedback in front of the group (2) and role-play and feedback within the group (3). The group that trained with SP interviews gained the highest scores with regard to interview skills. Five studies [24, 42, 49–51] used videotape review for feedback. Kraan [24] investigated a graded teaching program of medical interviewing skills. Each year a different set of skills was highlighted. In the first years, basic interviewing skills, medical history-taking skills and psychosocial issues were emphasized. Effective exchange of information and difficult situations such as dealing with aggressive patients or sexual problems were topics for advanced learners. Each small group had both a physician and a behavioural scientist as facilitators. Ozcakar [42] found that students having both verbal and visual (videotape review) feedback were more successful than those having verbal feedback alone. Although self-assessment of the students did not improve significantly, feedback based on videotaped interviews was superior to the feedback given solely based on the observation of assessors. Hulsman [49] showed that students valued SP interviews, video observation and feedback as instructive and helpful to develop their own strengths and to identify certain kinds of behaviour to improve. Nestel and Kidd [50] used peer tutors and reported no differences regarding patient-centred interview skills between groups taught by peers and those taught by faculty. Von Lengerke [51] and Fortin [7] found that SP interviews were evaluated as one of the most effective teaching methods. Von Lengerke performed a pre-post comparison of students’ self-assessed competencies and had participants evaluate key teaching methods. In addition to history taking, disclosure of diagnosis was taught in this course. Fortin [7] focused on integrating patient-centred skills (listening, negotiating, responding to emotion empathetically, focusing the patient’s story) into a medical interview skills course. Mini-lectures, demonstrations by faculty and role-play preceded the SP interviews.

#### Using virtual patients

One RCT by Vash [53] reported on small groups working on virtual surgical patients in a computer lab. The patient was initially introduced to them, and then the students worked through eight sections including interview (chief complaint), medical history and review of systems. Students had to ask relevant questions by typing them. Students in the lab performed better than their colleagues in the control group, which had seen patients in the surgery clinic instead. Significant differences were only found in the history taking area.

#### Small group workshops including real patients

Four interventions [9, 10, 26, 54] provided real patients. Fischer [54] included real patient interviews at the end of a course including role-play and simulated patients as well. Students interviewed real patients and videotaped the interviews. One aspect of the intervention was that the students visited the real patients in their homes. The interviews were watched back in the classroom and the students received feedback from facilitators and group members. The authors reported a significant learning progress and improvement in taking a case history. Results of self-reported questionnaires corresponded well with the results of the Objective Structured Clinical Examination (OSCE). Windish [10] compared a communication skills course applying SPs to a control group interviewing inpatients. Students in the intervention group were better at establishing rapport and were able to list more psychosocial history items. Evans [26] used real patients in the context of a communication skills course. The authors applied lectures, role-play, SP interviews and discussion as well. All three studies made use of videotape review. Novack [9] included interviews with real patients in a course using lectures, role-play and discussion as well as textbooks with additional information. Students were supposed to follow a chronically ill patient for 1 year and after regular interviews, write up progress notes.

#### Creative approaches

##### Improvisational theatre

Watson [55], as well as Shochet [56], implemented elective courses including improvisational theatre techniques to improve specific communication skills. In Shochet’s study, students practised specific skills including listening, affirmation, non-verbal communication and other skills. Students discussed the relevance of these skills in communication with their patients. The authors showed that students felt more confident in their role as future physicians after the course and that they improved their ability to be flexible in communication styles and “respond in the moment”. Most students thought that the concepts that were addressed in the course were highly relevant to the care of patients. Students in Watson’s classes felt they became better listeners and observers.

#### Lego® simulation

Harding and D’Eon [57] implemented a Lego® simulation in their interactive lecture to improve patient-centred interviewing skills. Student volunteers took on the roles of doctor and patient. The doctor had to query the patient and through his responses replicate the patient’s Lego® construction without looking at it. The authors found this intervention helped preclinical students to concentrate on interviewing skills without being preoccupied with medical knowledge.

## Discussion

### Heterogeneity of interventions

One clear finding of the literature review is that the included studies applied very heterogeneous teaching methods and determined different core areas to teach. While some interventions focused on content or structure of the medical interview and imparted techniques on “how to ask the right questions”, others highlighted non-verbal communication skills, patient-centeredness and establishing rapport. There is no accordance on when in medical education certain skills should be taught, leading to interventions that were taught for students at very different levels of training. While some studies evaluated long existing training programmes extending over several semesters, others investigated innovative approaches sometimes lasting only a few hours.

Fourteen studies included medical students in the preclinical years, eight studies included students in the clinical years and one study included both. Authors of the studies investigating improvisational theatre and Lego® simulation presumed that preclinical students might especially benefit from creative approaches where no significant medical knowledge was required. Not being preoccupied with complicated clinical reasoning may facilitate history-taking exercises for this subgroup and enhance patient-centred approaches.

Heterogeneity might also be due to the context dependence of the medical interview itself. Goals of the included studies were to enable students to attain a set of basic knowledge and skills in the medical interview. But encounters with patients are highly complex events and no simple approach can do justice to all possible processes and challenges in such interactions. No single course can comprehensively address all the communication problems that a physician will encounter, nor will skills be effective in every imaginable clinical situation.

Most articles in the field of history taking don’t differentiate between interview skills, interpersonal skills and communication skills – this conceptual mixture also contributed to the heterogeneity of interventions. Very often, specific interpersonal and communication skills (e.g. nonverbal behaviour, communication of empathy) are taught within the context of medical interview courses. Maybe an exact separation of these terms and definitions is neither even possible nor desirable as there is a continuum from communication skills to interview skills to history taking.

### Assessment of history taking skills

Six different methods of assessing learning progress were applied in the included studies. Many studies used more than one of the following:

1. Self-evaluation questionnaires
2. Free-text response on what students learned from the workshop
3. Written examinations
4. Qualitative analysis of students’ reflections and write-ups
5. Assessment of (videotaped) interviews by either trained observers, SPs or student tutors, either using a checklist/validated measurement tool or just giving a global impression
6. OSCE-stations and assessment of the interviews by trained observers or SPs, using a checklist/validated measurement tool.

Studies with a higher MERSQI score (>11.5) mostly used the latter methods (numbers 4, 5, 6) of assessing learners’ progress. Very often, they combined different methods and had self-report course evaluation forms as well as formal assessments of students’ interviews with SPs.

### Findings from the MERSQI score

If articles are sub-divided by methodological quality, it becomes apparent that studies with a higher MERSQI score (>11,5) often report on small-group skills workshops using role-play, simulated patients, virtual patients and/or real patients. In these courses, teachers and group, sometimes also SPs or peer tutors, give feedback. Mostly, interviews are videotaped to facilitate and enhance feedback. Studies with a lower MERSQI score (<9) frequently apply a more traditional approach using demonstrations, theoretical sessions and self-study. As creative approaches also tend to achieve a lower MERSQI score, innovative approaches don’t seem to be associated with a better study quality. Experiential approaches (“learning by doing”, see Table 1) achieved the highest MERSQI scores. Differences in MERSQI scores are primarily explicable by implementation of control groups, objective assessment of (videotaped) interviews and use of assessment tools. Limitations of the MERSQI score could be that the scale is based on a quantitative experimental study design paradigm that might underestimate qualitative or observational studies. Reliance on the MERSQI score only might therefore be biased towards particular forms of research.

### Implications for future research

With regard to content, the included interventions were often innovative, mostly well-thought-out and substantiated. Many of them were descriptive studies that relied on students’ self-evaluation and didn’t provide evidence that the intervention was effective in improving history-taking skills. Though there is a well-established methodology for adequate evaluative research that should be used if the effectiveness of history-taking courses is to be properly determined, studies mostly lack baseline measurement, randomization, adequate control groups, external measurement, blinded raters or standardized measurement scales. Often self-developed assessment scales were used although proven scales for external assessment do exist (for example the History-Taking Rating Scale (HTRS) [28], the Maastricht History-taking and Advice Checklist (MAAS) [24] or the Brown Interviewing Checklist (BIC) [6]). And although essential elements of effective history taking courses were defined in the 80s and 90s [33, 34], there is still no evidence-based gold standard that could serve as control group for an innovative new approach. Of course innovative ideas should be described in articles to provoke and stimulate discussion with colleagues but there is still a need for substantiated not just experiential studies. Innovative new concepts must be welcomed, but they should be coupled with acceptable methodology to examine and demonstrate their effectiveness [40].

An effort should always be made to question if certain interventions provide a more significant improvement for certain groups of students. There may be circumstances that predispose students to require more specific interventions, for example a non-native speaker of a language may need training in appropriate phrasing of questions as well as non-verbal cues to be most effective at history taking.

### Implications for curriculum planners and medical teachers

Small group workshops including interview simulations (role-play, SP interviews, virtual patients) and interviews with real patients, followed by feedback and discussion, are widespread and have been most thoroughly investigated and reported on. Feedback using videotape review seems to be particularly successful in providing students with instructive techniques in history taking. Students in the early preclinical state might profit from creative approaches helping them to focus on the interview skills and not being preoccupied by attempts to make diagnoses beyond their abilities. There is no evidence on when history-taking workshops should take place in the curriculum. Some authors recommend implementing them in the clinical clerkships, others favour implementation in preclinical years. Curriculum planners should consider addressing the reported decline in history-taking skills over time when medical interviewing is taught early in the curriculum, especially concerning psychosocial issues. This might be achieved by implementing a long-term “communication skills” course or by offering booster sessions later in the clinical years.

### Limitations of this review

It is possible that our search strategy may have missed some papers, especially those published in different languages as we only included articles written in English or German. However, it is unlikely that we missed a substantial number of relevant publications, especially as this review covers such a long period. But more important than that, this review only included published studies while it is recognized that many training programs do teach history taking in a variety of ways world wide that may not be mentioned in this review as they have not been published.

## Conclusions

History taking is an essential skill of every physician and has to be taught in the course of their medical education. Today, there are many studies demonstrating that students can acquire interview skills by specific workshops. There seems to be little evidence noting the superiority of one specific method however, there is a broad scope of interventions that all seem to provide history taking skills. It is not known if the acquired skills can be generalized across situations or maintained over time.

Important formal goals for this research area are to meet acceptable methodological standards for evaluative research. External measurement of students’ skills – either by a clinician, a SP or student/peer tutor utilizing established proven scales – is an important objective for the evaluation of future methods of teaching history taking. Practical examinations involving SPs, especially OSCE stations, should be gold standard in assessing history taking skills.

## References

[1] Nichols LO, Mirvis DM (1998). Physician-patient communication: does it matter?. Tenn. Med..

[2] Davidoff F, Deutsch S, Egan KL, Ende J (1996). Who Has Seen A Blood Sugar? - Reflections on Medical Education. Edited by the American College of Physicians.

[3] Engel GE, Morgan WL (1973). Interviewing and patient care.

[4] Lipkin M, Branch W (1987). The medical interview and related skills. Office Practice of Medicine.

[5] Engel GL (1973). Enduring attributes of medicine relevant for the education of the physician. Ann Intern Med.

[6] Novack DH, Volk G, Drossman DA, Lipkin M (1993). Medical interviewing and interpersonal skills teaching in US medical schools. Progress, problems, and promise. JAMA.

[7] Fortin AH, Haeseler FD, Angoff N, Cariaga-Lo L, Ellman MS, Vasquez L, Bridger L (2002). Teaching pre-clinical medical students an integrated approach to medical interviewing: half-day workshops using actors. J Gen Intern Med.

[8] Hatem DS, Barrett SV, Hewson M, Steele D, Purwono U, Smith R (2007). Teaching the medical interview: methods and key learning issues in a faculty development course. J Gen Intern Med.

[9] Novack DH, Dube C, Goldstein MG (1992). Teaching medical interviewing. A basic course on interviewing and the physician-patient relationship. Arch Intern Med.

[10] Windish DM, Price EG, Clever SL, Magaziner JL, Thomas PA (2005). Teaching medical students the important connection between communication and clinical reasoning. J Gen Intern Med.

[11] Sanson-Fisher R, Maguire P (1980). Should skills in communicating with patients be taught in medical schools?. Lancet.

[12] Novack DH (1987). Therapeutic aspects of the clinical encounter. J Gen Intern Med.

[13] Peterson MC, Holbrook JH, Von Hales D, Smith NL, Staker LV (1992). Contributions of the history, physical examination, and laboratory investigation in making medical diagnoses. West J Med.

[14] Hampton JR, Harrison MJ, Mitchell JR, Prichard JS, Seymour C (1975). Relative contributions of history-taking, physical examination, and laboratory investigation to diagnosis and management of medical outpatients. Br Med J.

[15] Kassirer JP (1983). Teaching clinical medicine by iterative hypothesis testing. Let’s preach what we practice. N Engl J Med.

[16] Roshan M, Rao AP (2000). A study on relative contributions of the history, physical examination and investigations in making medical diagnosis. J. Assoc. Physicians India.

[17] Sandler G (1980). The importance of the history in the medical clinic and the cost of unnecessary tests. Am Heart J.

[18] Bird J, Cohen-Cole SA (1990). The three-function model of the medical interview. An educational device. Adv Psychosom Med.

[19] Rezler AG, Woolliscroft JA, Kalishman SG (1991). What is missing from patient histories?. Med. Teach..

[20] Smith RC (2002). Patient-centered interviewing: an evidence-based method.

[21] Rosenberg EE, Lussier MT, Beaudoin C (1997). Lessons for clinicians from physician-patient communication literature. Arch Fam Med.

[22] Lazare A, Putnam S, Lipkin M (1995). Three functions of the medical interview. The Medical Interview.

[23] Engel GL (1982). What if music students were taught to play their instruments as medical students are taught to interview?. Pharos Alpha Omega Alpha Honor Med Soc..

[24] Kraan HF, Crijnen AA, de Vries MW, Zuidweg J, Imbos T, Van der Vleuten CP (1990). To what extent are medical interviewing skills teachable?. Med. Teach..

[25] Pfeiffer C, Madray H, Ardolino A, Willms J (1998). The rise and fall of students’ skill in obtaining a medical history. Med Educ.

[26] Evans BJ, Coman GJ, Goss B (1996). Consulting skills training and medical students’ interviewing efficiency. Med Educ.

[27] Evans BJ, Sweet B (1993). Consulting-skills training to improve medical students’ diagnostic efficiency. Acad.A Med..

[28] Evans BJ, Sweet B, Coman GJ (1993). Behavioural assessment of the effectiveness of a communication programme for medical students. Med Educ.

[29] Schildmann J, Kampmann M, Schwantes U (2004). Teaching courses on aspects of medical history taking and communication skills in Germany: a survey among students of 12 medical faculties. Z Arztl Fortbild Qualitatssich.

[30] Hargie O, Dickson D, Boohan M, Hughes K (1998). A survey of communication skills training in UK schools of medicine: present practices and prospective proposals. Med Educ.

[31] Assiciation of American Colleges (1998). Learning Objectives for Medical Student Education: Guidelines for Medical Schools (MSOP Report).

[32] General Medical Council (1991). Tomorrow’s Doctors: Recommendations on Undergraduate Medical Education.

[33] Maguire P (1990). Can communication skills be taught?. Br J Hosp Med.

[34] Simpson M, Buckman R, Stewart M, Maguire P, Lipkin M, Novack D, Till J (1991). Doctor-patient communication: the Toronto consensus statement. BMJ.

[35] Liberati A, Altman DG, Tetzlaff J, Mulrow C, Gotzsche PC, Ioannidis JP, Clarke M, Devereaux PJ, Kleijnen J, Moher D (2009). The PRISMA statement for reporting systematic reviews and meta-analyses of studies that evaluate health care interventions: explanation and elaboration. PLoS Med.

[36] Moher D, Liberati A, Tetzlaff J, Altman DG, Group P (2009). Preferred reporting items for systematic reviews and meta-analyses: the PRISMA statement. PLoS Med.

[37] Henwood PG, Altmaier EM (1996). Evaluating the effectiveness of communication skills training: a review of research. Clin. Perform. Qual. Health Care.

[38] Aspegren K (1999). BEME Guide No. 2: Teaching and learning communication skills in medicine-a review with quality grading of articles. Med. Teach..

[39] Reed DA, Cook DA, Beckman TJ, Levine RB, Kern DE, Wright SM (2007). Association between funding and quality of published medical education research. JAMA.

[40] Sanson-Fisher R, Fairbairn S, Maguire P (1981). Teaching skills in communication to medical students--a critical review of the methodology. Med Educ.

[41] Mukohara K, Kitamura K, Wakabayashi H, Abe K, Sato J, Ban N (2004). Evaluation of a communication skills seminar for students in a Japanese medical school: a non-randomized controlled study. BMC Med. Educ..

[42] Ozcakar N, Mevsim V, Guldal D, Gunvar T, Yildirim E, Sisli Z, Semin I (2009). Is the use of videotape recording superior to verbal feedback alone in the teaching of clinical skills?. BMC Public Health.

[43] Utting MR, Campbell F, Rayner C, Whitehouse CR, Dornan TL (2000). Consultation skills of medical students before and after changes in curriculum. J R Soc Med.

[44] Peltier D, Regan-Smith M, Wofford J, Whelton S, Kennebecks G, Carney PA (2007). Teaching focused histories and physical exams in ambulatory care: a multi-institutional randomized trial. Teach. Learn. Med..

[45] Losh DP, Mauksch LB, Arnold RW, Maresca TM, Storck MG, Maestas RR (2005). Teaching inpatient communication skills to medical students: an innovative strategy. Acad Med.

[46] Wiecha JM, Gramling R, Joachim P, Vanderschmidt H (2003). Collaborative e-learning using streaming video and asynchronous discussion boards to teach the cognitive foundation of medical interviewing: a case study. J. Med. Internet Res..

[47] Battles JB, Sprankell SJ, Carpenter JL, Bedford JA, Kirk LM (1992). Developing a support system for teaching and assessing clinical competence. J. Biocommun..

[48] Eoaskoon W, Sumawong V, Silpakit C (1996). Evaluation of training medical students in patient-interviewing skills by three modes of learning. J Med Assoc Thai.

[49] Hulsman RL, Harmsen AB, Fabriek M (2009). Reflective teaching of medical communication skills with DiViDU: assessing the level of student reflection on recorded consultations with simulated patients. Patient Educ Couns.

[50] Nestel D, Kidd J (2003). Peer tutoring in patient-centred interviewing skills: experience of a project for first-year students. Med. Teach..

[51] von Lengerke T, Kursch A, Lange K (2011). The communication skills course for second year medical students at Hannover Medical School: An evaluation study based on students’ self-assessments. GMS Z Med Ausbild.

[52] Yedidia MJ, Gillespie CC, Kachur E, Schwartz MD, Ockene J, Chepaitis AE, Snyder CW, Lazare A, Lipkin M (2003). Effect of communications training on medical student performance. JAMA.

[53] Vash JH, Yunesian M, Shariati M, Keshvari A, Harirchi I (2007). Virtual patients in undergraduate surgery education: a randomized controlled study. ANZ J Surg.

[54] Fischer T, Chenot J-F, Kleiber C, Kochen MM, Simmenroth-Nayda A, Staats H, Herrmann-Lingen C (2005). Kurs “ärztliche Basisfertigkeiten” - Evaluation eines primärärztlich orientierten Unterrichtskonzepts im Rahmen der neuen Approbationsordnung. GMS Z Med Ausbild.

[55] Watson K (2011). Perspective: Serious play: teaching medical skills with improvisational theater techniques. Acad Med.

[56] Shochet R, King J, Levine R, Clever S, Wright S (2013). ‘Thinking on my feet’: an improvisation course to enhance students’ confidence and responsiveness in the medical interview. Educ. Prim. Care.

[57] Harding SR, D’Eon MF (2001). Using a Lego-based communications simulation to introduce medical students to patient-centered interviewing. Teach. Learn. Med..

